# A Novel Six-mRNA Signature Predicts Survival of Patients With Glioblastoma Multiforme

**DOI:** 10.3389/fgene.2021.634116

**Published:** 2021-03-11

**Authors:** Zhentao Liu, Hao Zhang, Hongkang Hu, Zheng Cai, Chengyin Lu, Qiang Liang, Jun Qian, Chunhui Wang, Lei Jiang

**Affiliations:** ^1^Department of Neurosurgery, Changzheng Hospital, Naval Medical University, Shanghai, China; ^2^Department of Neurosurgery, No. 988 Hospital of Joint Logistic Support Force, Zhengzhou, China; ^3^Department of Orthopaedic Oncology, Changzheng Hospital, Naval Medical University, Shanghai, China; ^4^Department of Pharmacy, Changhai Hospital, Naval Medical University, Shanghai, China; ^5^Department of Neurosurgery, Tongji Hospital, Shanghai Tong Ji University School of Medicine, Shanghai, China

**Keywords:** glioblastoma multiforme, six-mRNA signature, LASSO, prognosis, biomarkers

## Abstract

Glioblastoma multiforme (GBM) is a devastating brain tumor and displays divergent clinical outcomes due to its high degree of heterogeneity. Reliable prognostic biomarkers are urgently needed for improving risk stratification and survival prediction. In this study, we analyzed genome-wide mRNA profiles in GBM patients derived from The Cancer Genome Atlas (TCGA) and Gene Expression Omnibus (GEO) databases to identify mRNA-based signatures for GBM prognosis with survival analysis. Univariate Cox regression model was used to evaluate the relationship between the expression of mRNA and the prognosis of patients with GBM. We established a risk score model that consisted of six mRNA (AACS, STEAP1, STEAP2, G6PC3, FKBP9, and LOXL1) by the LASSO regression method. The six-mRNA signature could divide patients into a high-risk and a low-risk group with significantly different survival rates in training and test sets. Multivariate Cox regression analysis confirmed that it was an independent prognostic factor in GBM patients, and it has a superior predictive power as compared with age, IDH mutation status, MGMT, and G-CIMP methylation status. By combining this signature and clinical risk factors, a nomogram can be established to predict 1-, 2-, and 3-year OS in GBM patients with relatively high accuracy.

## Background

Glioblastoma, also known as glioblastoma multiforme (GBM), is the most common primary malignant cancer involving the central nervous system (CNS), characterized by aggressive invasiveness and an infiltrative growth pattern (Lapointe et al., 2018; Lee et al., 2018). According to the Central Brain Tumor Registry of the United States (CBTRUS), GBM accounts for 14.9% of all primary brain tumors and 55.4% of all gliomas (Alexander and Cloughesy, 2017). Current standard treatment for GBM patients comprises maximal safe surgical resection, concurrent chemo-radiotherapy, and adjuvant chemotherapy with temozolomide (TMZ) (Laug et al., 2018). Despite recent advances in multi-modality strategies and individualized therapies, GBM patients usually have a dismal prognosis, with a median overall survival (OS) of less than 15 months (Lapointe et al., 2018).

The 2007 World Health Organization (WHO) classification system has been used for glioma classification over the past decade (Fuller and Scheithauer, 2007). This histological classification and grading system was largely based on visual criteria alone and fails to accurately evaluate the clinical outcomes of GBM patients. Actually, GBM patients with indistinguishable histopathological features can have different clinical outcomes. Accordingly, numerous efforts have been undertaken to characterize the underlying pathological molecular mechanisms and identify reliable prognostic markers for a precise prediction in GBM patients. More recently, some biomarkers including loss of 1p19q chromosome, methylation of the O6-methylguanine DNA-methyltransferase (MGMT), and iso-citrate dehydrogenases 1/2 (IDH1/2) have been found to be closely related to the therapy sensitivity and prognosis (Smits and van den Bent, 2017; Feyissa et al., 2019; Radke et al., 2019). MGMT promoter region was recognized to have a predictive value for the efficacy of TMZ-based chemotherapy, and mutations in the genes encoding for iso-citrate dehydrogenases 1/2 (IDH1/2) could predict a relatively long-term survival of GBM patients (Feyissa et al., 2019; Radke et al., 2019). In the 2016 updated version of the WHO classification system, molecular information has been integrated into pathological diagnosis for further subgroup stratification (Louis et al., 2016). However, due to the biological heterogeneity of GBM, individual biomarkers alone are unable to predict the therapy sensitivity and survival of GBM patients. In this regard, there is an urgent need to identify more effective tumor biomarkers for risk stratification and prognosis prediction.

RNA sequencing (RNA-Seq) is the most commonly used high-throughput technology for transcriptome profiling and offers more sensitive and accurate measurement of gene expression as compared with traditional gene expression arrays (Wang et al., 2009). The Cancer Genome Atlas (TCGA) has accrued RNA-Seq-based transcriptome data across various cancer types and thus provides a robust platform to systematically analyze the large-scale RNA-Seq data (Peng et al., 2015). A comprehensive characterization of gene expression changes in GBM will provide a large number of new potential predictive and prognostic molecular markers. In this study, we retrieved mRNA expression profiles from a large number of GBM patients and analyzed them by re-purposing the publicly available TCGA database to identify an effective signature of mRNAs for predicting survival of GBM patients.

## Methods

### TCGA GBM and GSE108474 Datasets Download and Processing

The TCGA GBM data (Level 3 RNA-Seq) of 174 individuals with clinical information were extracted from the TCGA (GDC) database, including data from 169 GBM tissues and 5 normal brain tissues. The exclusion criteria are as follows: (1) GBM was ruled out by histological diagnosis, (2) the existence of another malignancy with GBM, and (3) patient death due to unrelated causes. Only patients who were followed up for longer than a month were included in the subsequent analysis. Finally, 152 GBM patients were included in this study. Seventy percent of the TCGA patients were randomly selected as the training set and the remaining 30% of the patients were selected as the test set. As the data were downloaded from the public database, ethical approval was not applicable in this case. The data processing procedures met the policies of TCGA data access and human subject protection^1^. GSE108474 from the GEO database was conducted through GPL (Affymetrix Human Genome U133 Plus 2.0 Array)^2^. A total of 210 GBM samples were selected from the GSE108474 database. The GEO data were also downloaded from the public database platform. Count data from the TCGA set and GSE108474 set were integrated into expression matrix, which were converted to TPM and standardized to *Z* score data for subsequent model analysis.

### Identification of Differentially Expressed mRNAs in GBM

Three bioconductor software analysis packages—Deseq2, limma + voom, and edgerR—are used to analyze the difference of mRNA expression read count data. The expression differences were characterized by logFC (log2 fold change). mRNAs with | logFC| > 1 (*P* < 0.05) were considered differentially expressed mRNAs and selected for further analysis.

### Identification of mRNAs Significantly Associated With OS and Prognostic Signature Construction in GBM

The semi-supervised method combining both the gene expression data and clinical data was used to identify candidate mRNAs with a prognostic value. Univariate Cox regression analysis was conducted to identify common mRNAs related to OS within each of the subgroups stratified by the IDH status. Within each group of clinical characteristics, the patient subclasses represented non-overlapping sets. Common mRNAs associated with OS in at least two independent subgroups were selected for the subsequent study, using an HR > 1 or HR < 1 with *P* < 0.05 as the cutoff.

### Definition of the Prognostic Risk Model and ROC Curve Analysis

The TCGA dataset “caret” package was randomly divided into a training set and a testing set. An importance score was calculated by the supervised principal component method and assigned to each mRNA. Tenfold cross-validation was used to estimate the optimal feature threshold in supervised principal components and select significant mRNAs. The linear mRNA signature prognostic model was developed based on the supervised principal component method. The mRNA expression level was expressed as the *Z* score. The prognostic score was calculated as follows: Prognostic score = (0.058 × Expression AACS) + (0.015 × Expression STEAP1) + (0.009 × Expression STEAP2) + (0.039 × Expression G6PC3) + (0.014 × Expression FKBP9) + (0.067 × Expression LOXL1). Then, the prognostic scores were computed for the 152 patients using our mRNAs prognostic model. The optimal cutoff value of the prognostic score was decided in the ROC (receiver operating characteristic) curve analysis for predicting 1-, 2-, and 3-year survival of the training set. The OS curves were evaluated using the Kaplan–Meier (KM) and log-rank method. Time-dependent ROC curves were used to evaluate the predictive power of the mRNA signature model. All analyses were performed using the R (version 3.5.2) and Bioconductor (version 3.9).

### Survival Analysis

The differences in clinical features including sex, age, IDH1, MGMT, and the survival status between the training set, internal testing set, and independent validation set were analyzed using the chi-square test. A univariate Cox model was performed to investigate the relationship between the continuous expression level of each DEmRNA and OS and for preliminary screening of clinical variables that were correlated with OS of the GBM patients. Hazard ratios (HRs) and *P*-values from univariate Cox regression analysis were used to identify candidate survival-related DEmRNAs. DEmRNAs with HR for death > 1 were defined as high-risk RNAs, and those with HR < 1 were defined as protective RNAs. The common DEmRNAs meeting the criterion of *P*-value < 0.05 were selected as survival-related DEmRNAs and further analyzed in LASSO regression to identity the most powerful prognostic markers. Finally, a multi-mRNA-based classifier was constructed for predicting OS. To quantify the risk of OS, a standard form of risk score (RS) for each GBM patient was calculated in combination with the relative expression levels of the mRNAs.

### Statistical Analysis

Mann–Whitney *U*-test and χ^2^-test were used to compare the correlation between continuous variables and classified variables between the training data and test data, respectively. The independent prognostic variables of OS were screened by univariate and multivariate regression analyses. L1-LASSO penalization was carried out using “glmnet” and “pliable” software packages. KM method was used to draw the survival curve based on survminer software package, and log-rank test was used to compare it. Using “rms” software package, Cox’s regression coefficient was calculated to establish the risk assessment formula and mRNA nomogram. Knowing that time-dependent ROC curve analysis is widely used in biomedical reports to evaluate the prediction accuracy of six mRNA signatures, we used the “timeROC” software package to analyze the time-dependent ROC curve. The volcanoes and heat maps were drawn by using the “ggpolt2” software package of the R software. All other statistical tests were carried out with R software version 3.5.2 and the corresponding basic software packages. The value of *P* < 0.05 was determined to be statistically significant.

## Results

### Baseline Characteristics of the Patients and Analysis Flow

**FIGURE 1:**
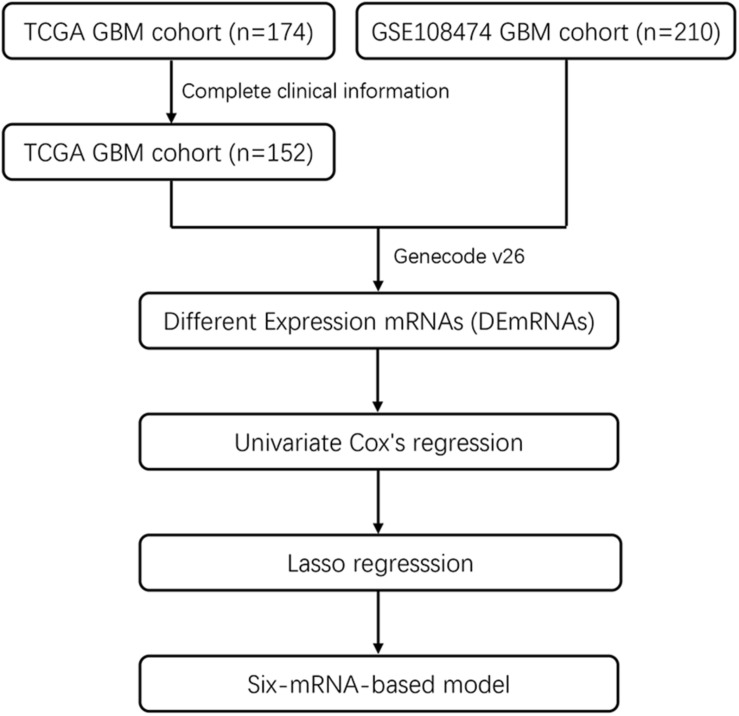
Flowchart showing steps involved in identification of multi-mRNA-based prognostic signature in GBM.

Included in this study were 152 GBM patients from TCGA database and 210 GBM patients from the GEO database (GSE108474). The detailed baseline characteristics of the training set and test set are listed in Table 1 from the TCGA database. The results showed no significant difference in the baseline characteristics between the two sets (all *P* > 0.05). A total of 210 GBM patients from the GEO database make up an independent validation set. Our research process is shown in Figure 1.

**TABLE 1:** Baseline characteristic of study patients.

Variables	Training date	Test date	Overall	*P*-value
No. of patients	107	45	152	
Age (years), *N* (%)				1
Mean (SD)	59.2 (13.4)	59.8 (13.4)	59.6 (13.3)	
Median [Min, Max]	59.0 [21.0, 79.0]	62.0 [21.0, 85.0]	60.0 [21.0, 85.0]	
Age (years), *N* (%)				0.668
< 50	11 (24.4%)	22 (20.6%)	33 (21.7%)	
≥ 50	34 (75.6%)	85 (79.4%)	119 (78.3%)	
Sex, *N* (%)				0.143
Female	20 (44.4%)	34 (31.8%)	54 (35.5%)	
Male	25 (55.6%)	73 (68.2%)	98 (64.5%)	
KPS type, *N* (%)				
<70	9 (20.0%)	23 (21.5%)	32 (21.1%)	0.921
≥70	26 (57.8%)	58 (54.2%)	84 (55.3%)	
Unknown	10 (22.2%)	26 (24.3%)	36 (23.7%)	
IDH1 type, *N* (%)				0.486
MT	2 (4.4%)	7 (6.5%)	9 (5.9%)	
WT	40 (88.9%)	97 (90.7%)	137 (90.1%)	
Unknown	3 (6.7%)	3 (2.8%)	6 (3.9%)	
CIMP type, *N* (%)				0.787
G-CIMP	3 (6.7%)	6 (5.6%)	9 (5.9%)	
Non-G-CIMP	41 (91.1%)	100 (93.5%)	141 (92.8%)	
Unknown	1 (2.2%)	1 (0.9%)	2 (1.3%)	
MGMT type, *N* (%)				0.864
Methylated	18 (40.0%)	38 (35.5%)	56 (36.8%)	
Unmethylated	18 (40.0%)	45 (42.1%)	63 (41.4%)	
Unknown	9 (20.0%)	24 (22.4%)	33 (21.7%)	
Race type, *N* (%)				0.448
Black or African. American	3 (6.7%)	6 (5.6%)	9 (5.9%)	
White	42 (93.3%)	95 (88.8%)	137 (90.1%)	
Asian	0 (0%)	5 (4.7%)	5 (3.3%)	
Unknown	0 (0%)	1 (0.9%)	1 (0.7%)	
Status, *N* (%)				0.825
Alive	9 (20.0%)	20 (18.7%)	29 (19.1%)	
Dead	36 (80.0%)	87 (81.3%)	123 (80.9%)	
OS time				0.883
Mean (SD)	461 (427)	439 (364)	445 (382)	
Median [Min, Max]	394 [64.0, 2680]	382 [33.0, 2130]	383 [33.0, 2680]	

### Candidate OS-Related mRNAs of GBM Patients in the TCGA Cohort

**FIGURE 2:**
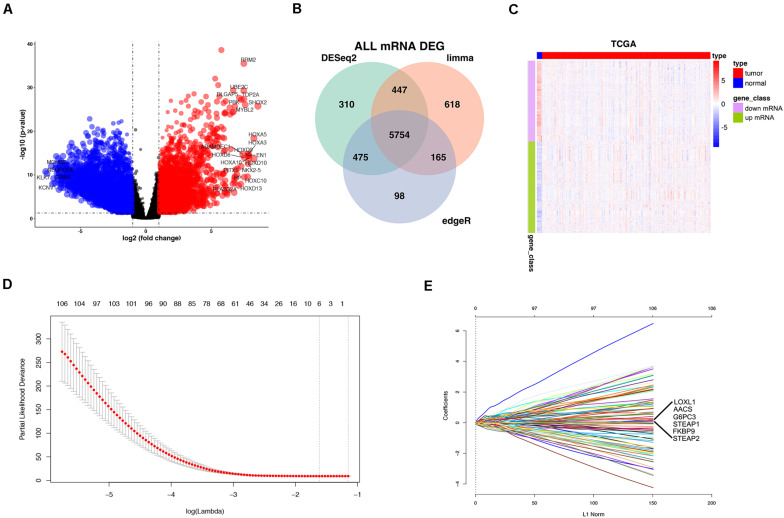
Differentially expressed mRNAs (DEmRNAs) in GBM vs. normal tissues. Volcano Plot: The colorized points in scatter plot represent the DEmRNAs with statistical significance (*P*-value < 0.05, |logFC| > 1) **(A)**. Venn Plot visualizing the DEmRNAs which was screened by limma Deseq2 and edgeR **(B)**. Two-way hierarchical clustering of 152 tumor tissues and 5 normal tissues with the 2114 differentially expressed RNAs using Euclidean distance and average linkage clustering **(C)**. mRNA selection using the least absolute shrinkage and selection operator (LASSO) binary COX regression model. Tuning parameter (λ) selection in the LASSO model **(D)**. LASSO coefficient profiles **(E)**.

In the TCGA database, 5754 differentially expressed mRNAs [false detection rate < 0.05 and log2 fold change (Log2fc) ≥ 1] were identified by using the expression profiles of 19,781 mRNAs in 152 cases of GBM and 5 cases of normal brain tissues. These 5754 mRNA volcanoes were visualized through the “ggplot2” package of R software (Figures 2A–C). These 5754 differentially expressed mRNAs were considered to be the potential prognostic markers of GBM patients, in which the expression of 3054 mRNAs was up-regulated and that of 2700 mRNAs was down-regulated. To filtrate out the mRNAs associated with OS, the Cox’s regression and LASSO regression analyses (lambda parameter selection 1SE) were performed in sequence (Figures 2D,E). Finally, six mRNAs related to OS were selected, including AACS, STEAP1, STEAP2, G6PC3, FKBP9, and LOXL1 (Table 2).

**TABLE 2:** Six prognostic mRNAs associated with OS in the training set.

Name	Coefficient	Type	Down/up-regulated	HR	95% CI	*P-*value
AACS	0.058	Risky	Down	1.132	1.028–1.126	0.011*
STEAP1	0.015	Risky	Up	1.012	1.003–1.021	0.012*
STEAP2	0.009	Risky	Down	1.08	1.035–1.126	<0.001*
G6PC3	0.039	Risky	Up	1.012	1.005–1.019	<0.001*
FKBP9	0.014	Risky	Up	1.003	1.001–1.005	0.004*
LOXL1	0.067	Risky	Up	1.009	1.004–1.013	<0.001*

*P < 0.05 was considered statistically significant.

### Development of the Risk Score Formula and Six-mRNA-Based Prognostic Model

**FIGURE 3:**
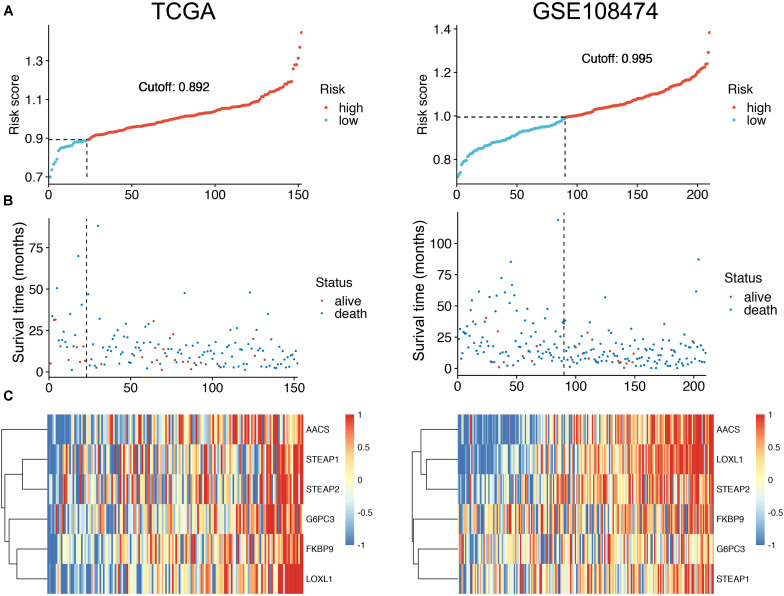
The distribution of risk scores **(A)**. Patient survival time and status. The black dotted line represents the optimum cutoff dividing patients into low-risk and high-risk groups **(B)**. The expression heat map of the mRNAs in a prognostic classifier **(C)**.

**FIGURE 4:**
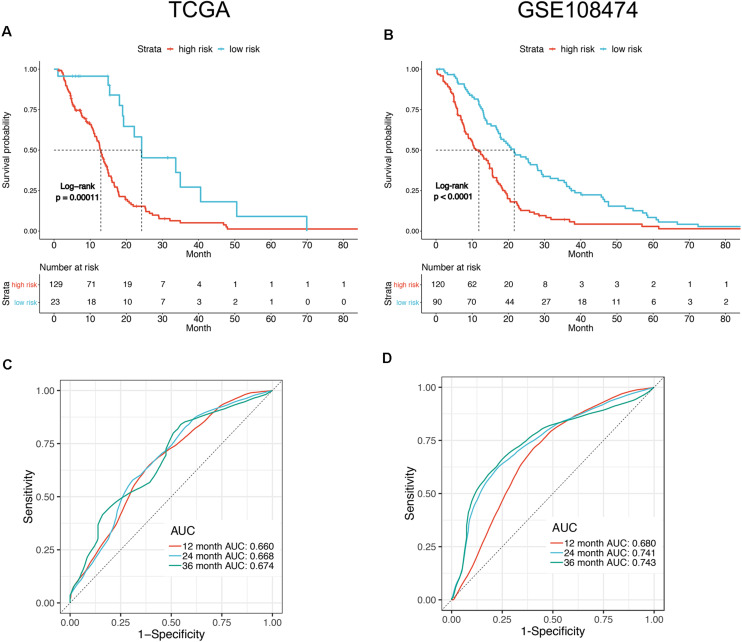
Survival analysis of the patients divided by prognostic mRNA in high and low risk. Kaplan–Meier curves of overall survival in TCGA **(A)** and GSE108474 **(B)** datasets showed poor survival for patients with a high-risk score. ROC curve for 1, 2, and 3 year survival prediction by the six-gene signature in TCGA **(C)** and GSE108474 **(D)** datasets.

To facilitate the application of identified OS-related mRNAs in clinical practice, the risk score of each patient was calculated using the following equation: Risk score = (0.058 × Expression AACS) + (0.015 × Expression STEAP1) + (0.009 × Expression STEAP2) + (0.039 × Expression G6PC3) + (0.014 × Expression FKBP9) + (0.067 × Expression LOXL1). According to the risk score, Youden index (Hughes, 2015) was set to the cutoff value, based on which GBM patients were categorized into a low-risk group and a high-risk group (Figure 3A). The survival status of GBM patients in the low- and high-risk groups was obvious in both cohorts (Figure 3B). The heat map showed the expression of six mRNAs (AACS, STEAP1, STEAP2, G6PC3, FKBP9, and LOXL1) in the samples (Figure 3C). In addition, compared with the low-risk group, KM survival analysis showed that the prognosis of GBM patients in the high-risk group was significantly worse than that in the low-risk group (training and test set in TCGA database *P* < 0.00011, validation set of GEO database *P* < 0.0001) (Figures 4A,B). To evaluate the prognostic prediction performance of GBM patients based on the six-mRNA signature model, we compared the AUC values at different time points to draw the survival ROC curve. The AUC value of the primary set and the validation set at 1, 2, and 3 years was 0.660, 0.668, and 0.674, and 0.680, 0.741, and 0.743, respectively (Figures 4C,D).

To analyze risk factors affecting the prognosis of GBM patients, univariate Cox’s analysis was performed and the result showed that the prognosis of GBM patients was associated with age, IDH1 mutation, MGMT methylation, CIMP methylation, and the six-mRNA signature, but not with gender and KPS (Karnofsky) score. Subsequent multivariate analysis showed that the six-mRNA signature was an independent prognostic survival factor in GBM patients (Table 3).

**TABLE 3:** Univariate and multivariate Cox’s regression analyses in the training set.

Variables		Patients (*N*)	Coefficient	Univariate analysis			Multivariate analysis		
					
				HR	95% CI	*P*-value	HR	95% CI	*P*-value
Age	≥50/<50	119/33	0.579	1.784	1.13–2.818	0.013*	1.143	0.692–5.563	0.601
Gender	Female/Man	54/98	0.062	1.064	0.732–1.547	0.745			
KPS	≥70/<70	108/44	–0.084	0.915	0.611–1.368	0.663			
IDH1	Unknown/Mut	6/9	0.217	1.242	0.176–8.758	0.828			
	WT/Mut	137/9	1.176	3.242	1.317–7.979	0.011*	1.324	0.234–7.493	0.751
MGMT	Methylated/Unknown	33/63	–0.306	0.736	0.458–1.182	0.205			
	Methylated/Unmethylated	56/63	–0.474	0.622	0.408–0.949	0.028*	0.681	0.444–1.045	0.079
CIMP	Unknown/non-G-CIMP	2/141	–17.360	0.000	0–inf	0.995			
	G-CIMP/non-G-CIMP	9/141	–1.196	0.303	0.111–0.822	0.019*	0.835	0.138–5.031	0.844
Six-mRNA signature	High/Low	129/23	1.048	2.850	1.621–5.02	0.00028*	2.901	1.513–5.563	0.001*

*P < 0.05 was considered statistically significant.

### Predictive Ability of the Six-mRNA-Based Model in Different Risk Stratification

**FIGURE 5:**
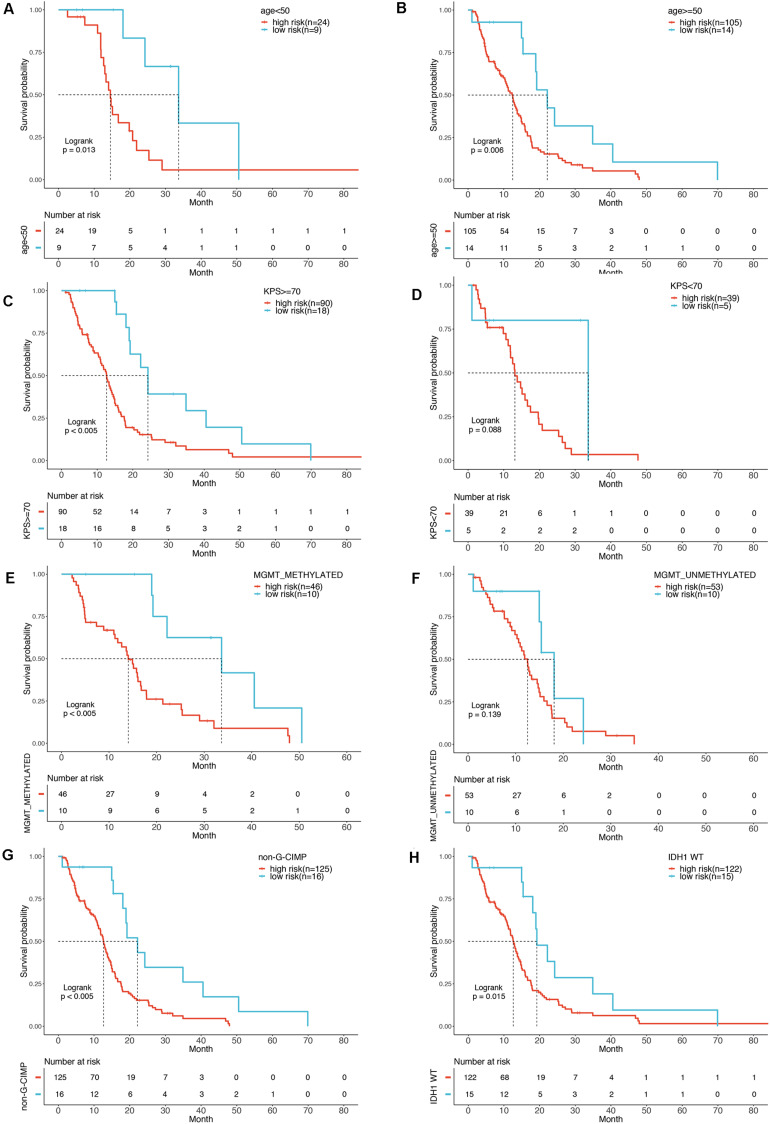
Stratification analysis by different clinical variables. Meier curve analysis of overall survival in high- and low-risk group for younger (age < 50) **(A)** and older patients (age ≥ 50) **(B)**. KPS ≥ 70 **(C)**, and < 70 **(D)**. MGMT methylated status as methylated **(E)** and unmethylated **(F)**. G-CIMP methylated **(G)**. IDH1 mutation status as WT **(H)**.

To show whether the six-mRNA-based model could predict the OS time of patients, clinical risk factors including age, KPS score, IDH1 mutation status, MGMT methylation status, and CIMP methylation status in GBM patients were stratified. In patients with age ≧ 50 years (*P* = 0.006), age < 50 years (*P* = 0.013), MGMT methylated (*P* < 0.005), KPS ≧ 70 (*P* < 0.005), CIMP unmethylated (*P* < 0.005), and IDH1 WT (*P* = 0.015), OS of GBM patients in the low-risk group was significantly better than that in the high-risk group. However, in patients with KPS < 70 (*P* = 0.088) and MGMT unmethylated (*P* = 0139), there was no significant difference in OS between the low-risk and high-risk groups (Figures 5A–H).

**FIGURE 6:**
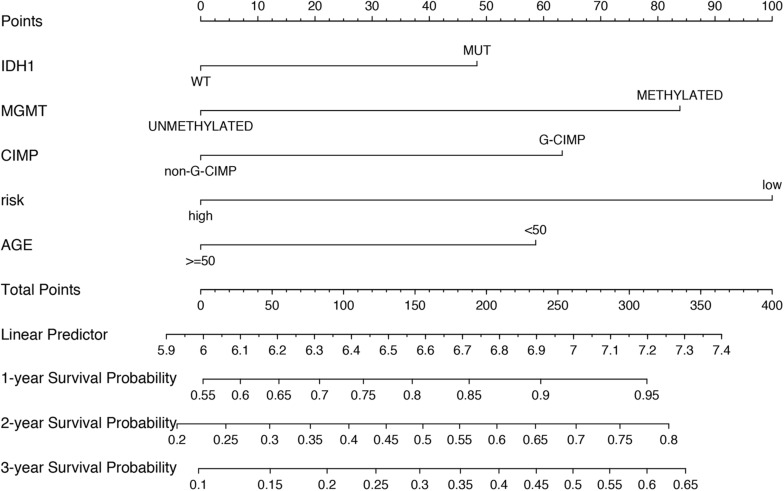
Nomograms to predict 1, 2, and 3 year survival probability in GBM. Total points were obtained by adding up the corresponding points of each individual covariate on the points scale.

To build a convenient and sensitive predictive tool for clinical use, a new prognostic model based on the six-mRNA model was established by combining signature and four clinical risk factors (age, IDH1 status, MGMT status, and CIMP status) to predict 1-, 2-, and 3-years OS in GBM patients. The results showed that the six-mRNA signature and MGMT methylated status contributed most to OS in 1, 2, and 3 years, followed by the CIMP methylated status, patient age, and the IDH1 mutation status in six-mRNA-based nomograms. Each variable obtained a nomogram score on the point scale. By calculating the total score of the nomogram, we easily obtained the nomogram prediction probability of each patient for 1-, 2-, and 3-years OS (Figure 6).

### Comparison With Other Prognostic Factors

**FIGURE 7:**
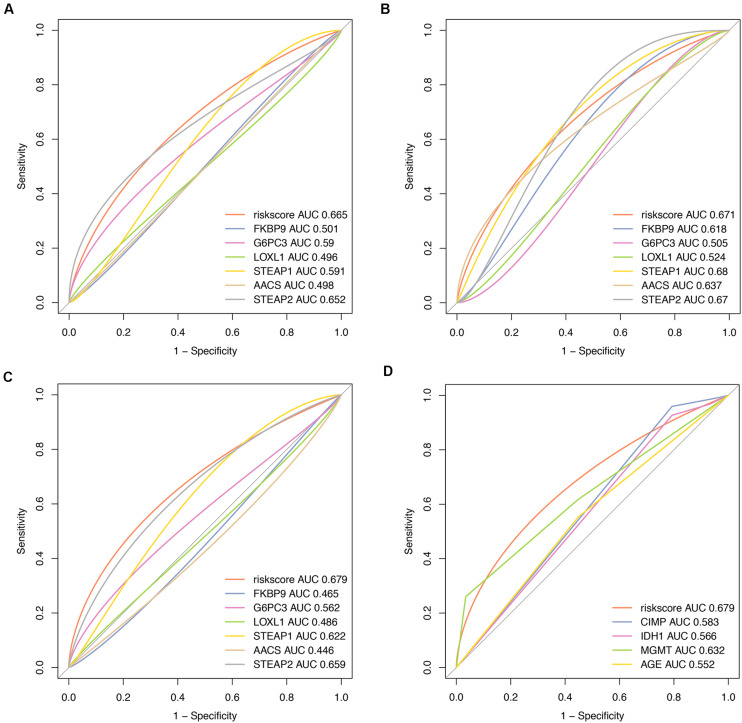
Comparisons of prognostic accuracy at 1 year using the time-dependent receiver operating characteristic curve in the six-mRNA signature with a single mRNA from training date **(A)**, test date **(B)**, TCGA date **(C)** separately, and the six-mRNA-based prognostic model with age, IDH1 status, MGMT methylated status, and G-CIMP methylation in GBM **(D)**.

To compare the predictive accuracy of different prognostic factors, ROC analysis showed that the six-miRNA signature had higher prognostic accuracy than mRNA alone (Figures 7A–C). More importantly, the accuracy of six-mRNA signature prediction was also significantly better than that of clinical risk factors such as age, IDH1 mutation status, MGMT methylation, and G-CIMP methylation status (Figure 7D).

## Discussion

Transcriptional aberrations play a crucial role in the complex regulatory network of glioblastoma and are of great importance for the prognosis of this deadly cancer (Bian et al., 2018). Previous studies mainly focused on the function of individual differently expressed mRNAs, which may not be sufficient to clarify the underlying mechanism of tumorigenesis and predict the prognosis of GBM patients (Stoltz et al., 2015; Hu et al., 2017; Yu et al., 2017). Given the high molecular heterogeneity of GBM, integrating multiple biomarkers into a prediction model could improve the prognostic value as compared with a single biomarker. Such multiple-gene signature can be used in prognosis prediction and personalized therapy due to its great power and superiority of risk stratification in many cancers including GBM (Lai et al., 2019; Zuo et al., 2019). Based on this gene signature, patients can be divided into different risk groups so that more sophisticated or intensive targeted therapies could be selected accordingly.

Currently, many prognostic studies have sought to find GBM-specific genomic signatures based on massive genomic data generated by the large-scale cancer genomics projects such as the TCGA database. Yao et al. (2012) have introduced an algorithm using Cox proportional hazard regression and random re-sampling to identify functional gene sets linked to patient survival based on microarray gene expression datasets (TCGA and REMBRANDT). Another study conducted by Jing et al. (Han and Puri, 2018) found that the expression of interleukin-13 receptor α1 and α2 genes was associated with poor prognosis in GBM patients by using TCGA data. In addition, Wang et al. (2016) developed a signature with three genes that had prognostic values for patients with MGMT promoter-methylated GBM. However, it should be noted that the predictive power might be limited by the small number of individual mRNAs or specific tumor entities in these studies. Recently, Zuo et al. (2019) built an RNA-Seq-based six-gene signature to predict survival in patients with GBM by analyzing RNA-Seq-based gene expression profiles in the CGGA and TCGA databases. They found that this signature could divide patients into a high-risk or low-risk group with different OS and was independent of other clinicopathological features. However, the mRNAs in Zuo’s six-gene signature were not all screened from differentially expressed genes in glioma and normal brain tissues, which may limit its significance in revealing tumor heterogeneity. For this reason, we screened the differentially expressed genes by Cox proportional hazard regression in the present study and only chose GBM survival-related PCGs for further analysis in LASSO regression. Consequently, a six-gene signature was generated using gene expression data from two public databases and was validated in two cohorts of patients.

Various factors may affect the survival of GBM patients, including age, KPS score, extent of surgical removal, MGMT methylated, and the IDH1 mutation status. Our univariate analysis of the TCGA cohort showed that age, IDH1 mutation, MGMT methylated, and CIMP methylated were significantly associated with OS of GBM patients, which is consistent with numerous previous studies (Hegi et al., 2008; Cohen et al., 2013; de Souza et al., 2018). In our study, in GBM patients with age ≧50 years (*P* = 0.006), age <50 years (*P* = 0.013), MGMT methylated (*P* < 0.005), KPS ≧ 70 (*P* < 0.005), CIMP unmethylated (*P* < 0.005), and IDH1 WT (*P* = 0.015), OS of patients in the low-risk group was significantly better than that in the high-risk group. Furthermore, our subsequent multivariate analysis confirmed that this six-gene signature was an independent prognostic predictor of survival for GBM patients.

The six-mRNA signature identified in this study includes four up-regulated mRNAs (STEAP1, G6PC3, FKBP9, and LOXL1) and two down-regulated mRNAs (STEAP2 and AACS) with respect to their expression levels in GBM tissue samples. Among these genes, a few genes have established roles in cancers, and other genes might be potential new biomarkers for cancers. STEAP1 and STEAP2, the members of the human six-transmembrane epithelial antigen of the prostate (STEAP) protein family, are highly over-expressed in many different cancer entities including prostate, bladder, breast, colon, and lung carcinoma, Ewing’s sarcoma, anaplastic thyroid carcinoma, and malignant melanoma (Hubert et al., 1999; Valenti et al., 2009; Gomes et al., 2012). The high rate of co-expression of STEAP1 and 2 has been observed in cancer cell lines. However, the functional role of STEAP1 and STEAP2 in glioma has not yet been established. Acetoacetyl-CoA synthetase (AACS, acetoacetate-CoA ligase) plays an important role in cholesterol homeostasis and normal neuronal development and was found abundant in normal brain tissues (Ohgami et al., 2003). Knockdown of AACS in primary neurons decreased the expression of MAP-2 and NeuN, two known markers of neuronal differentiation (Hasegawa et al., 2012). G6PC3 is known to encode enzymes that have glucose-6-phosphatase activity, which is ubiquitously expressed in various tissues (Hayee et al., 2011). TP53 can reduce gluconeogenesis by down-regulating the expression of G6PC gene in colon and liver cancer cells and *in vivo*, thus implying an important regulatory relationship between TP53 and G6PC gene (Zhang et al., 2014). FKBP9 belongs to the FK506-binding protein (FKBP) family, which has peptidyl–prolyl cis–trans isomerases with the enzymatic function attributable to the FKBP domain (Jo et al., 2001). LOXL1, like other Lysyl oxidase (LOX) family members, has an established role in modifying the tumor microenvironment by crosslinking collagens and elastin in the extracellular matrix (Behmoaras et al., 2008). Increased LOXL1 was found in pancreatic ductal adenocarcinoma (Le Calve et al., 2016). In addition, the expression of LOXL1 was found to be correlated with T invasion, lymph node metastasis, and lymphatic and venous invasion in gastric cancer (Kasashima et al., 2014).

In adopting the prediction model of this study, the following limitations need to be considered. First of all, the biological functions and molecular mechanisms of the six mRNAs in the prediction model in glioma remain unclear and further researches are needed. Second, clinical data of postoperative intervention measures for GBM patients in TCGA and GEO databases are incomplete, such as radiotherapy and chemotherapy, so we cannot conduct a comprehensive analysis of OS. Third, the prediction model cannot effectively predict the patient OS with KPS <70 and MGMT demethylation between the low- and high-risk group. Therefore, multi-center, large-scale, prospective studies are needed to validate the predictive model in clinical practice.

## Conclusion

In this study, we have identified a six-gene signature for predicting survival of GBM patients by analyzing RNA-Seq gene expression profiles in the TCGA and GEO databases. This multiple-RNA-based signature could effectively stratify GBM patients into low- and high-risk groups with separate survival curves. Our multivariate analysis demonstrated that this six-gene signature is an independent prognostic predictor of survival of GBM patients. To the best of our knowledge, this is the first report about the prognostic value of the six RNAs (STEAP1, G6PC3, FKBP9, STEAP2, AACS, and LOXL1) in GBM, which may serve as new genetic clues for a better understanding about the development, progression, and recurrence of GBM.

## Data Availability Statement

Publicly available datasets were analyzed in this study. This data can be found here: http://gepia.cancer-pku.cnGSE108474.

## Author Contributions

ZL, HZ, and HH analyzed the data and wrote the manuscript. ZC and CL designed the study and performed data. QL and JQ prepared the figures and tables. All authors read and approved the final manuscript.

## Conflict of Interest

The authors declare that the research was conducted in the absence of any commercial or financial relationships that could be construed as a potential conflict of interest.

## Footnotes

1 http://cancergenome.nih.gov/publications/publicationguidelines

2 https://www.ncbi.nlm.nih.gov/geo/

## Abbreviations

GBM: Glioblastoma multiforme
OS: Overall survival
L1-LASSO: Least absolute shrinkage and selection operator
TCGA: The Cancer Genome Atlas
GEO: Gene Expression Omnibus
ROC: Receiver operating characteristic
AUC: Area Under Curve
KPS: Karnofsky
WT: wild type
MT: mutant type
IDH1: isocitrate dehydrogenase 1
MGMT: O6-methylguanine DNA-methyltransferase
CIMP: CpG Island Methylator Phenotype.
